# The impact of theory-based messages on COVID-19 vaccination intentions: a structured summary of a study protocol for a randomised controlled trial

**DOI:** 10.1186/s13063-021-05277-7

**Published:** 2021-04-29

**Authors:** Ben Young, Marie Kotzur, Lauren Gatting, Carissa Bonner, Julie Ayre, Alex McConnachie, Carys Batcup, Kirsten McCaffery, Ronan O’Carroll, Kathryn A. Robb

**Affiliations:** 1 University of Glasgow, Glasgow, UK; 2 University of Sydney, Sydney, Australia; 3 University of Stirling, Stirling, UK

**Keywords:** COVID-19, randomised controlled trial, protocol, vaccination, vaccine hesitancy, messaging, Necessity-Concerns Framework, illness beliefs, treatment beliefs

## Abstract

**Objectives:**

Uptake of vaccination against COVID-19 is key to controlling the pandemic. However, a significant proportion of people report that they do not intend to have a vaccine, often because of concerns they have about vaccine side effects or safety. This study will assess the impact of theory-based messages on COVID-19 vaccination intention, drawing on the Necessity-Concerns framework to address previously reported beliefs and concerns about COVID-19 vaccination, and assess whether hypothesised variables (illness coherence, perceived necessity and concerns) mediate change in vaccination intention.

**Trial design:**

Prospective, parallel two-arm, individually randomised (1:1) trial.

**Participants:**

Adults aged over 18 years, living in Scotland and not vaccinated for COVID-19. A quota sampling approach will be used with the aim of achieving a nationally representative sample on gender, region and ethnic group, with oversampling of individuals with no educational qualifications or with only school-level qualifications.

**Intervention and comparator:**

Intervention: Brief exposure to online text and image-based messages addressing necessity beliefs and concerns about COVID-19 vaccination.

Comparator: Brief exposure to online text and image-based messages containing general information about COVID-19 and COVID-19 vaccination.

**Main outcomes:**

Primary outcome: Self-reported intention to receive a vaccine for COVID-19 if invited, immediately post-intervention. Secondary outcomes: Self-reported COVID-19 illness coherence, perceived necessity of a COVID-19 vaccine and concerns about a COVID-19 vaccine, immediately post-intervention.

**Randomisation:**

Quasi-randomisation performed automatically by online survey software, by creating a variable derived from the number of seconds in the minute that the participant initiates the survey. Participants starting the survey at 0-14 or 30-44 seconds in the minute are allocated to the intervention and 15-29 or 45-59 seconds to the comparator.

**Blinding (masking):**

Participants will not be blinded to group assignment but will not be informed of the purpose of the study until they have completed the follow-up survey. Investigators will be blinded to allocation as all procedures will be undertaken digitally and remotely without any investigator contact with participants.

**Numbers to be randomised (sample size):**

A total of 1,094 will be randomised 1:1 into two groups with 547 individuals in each.

**Trial Status:**

Protocol version number 1.0, 26^th^ February 2021.

Recruitment status: Not yet recruiting, set to start April 2021 and end April 2021.

**Trial registration:**

ClinicalTrials.gov, NCT04813770, 24^th^ March 2021.

**Full protocol:**

The full protocol is attached as an additional file, accessible from the Trials website (Additional file 1). In the interest in expediting dissemination of this material, the familiar formatting has been eliminated; this Letter serves as a summary of the key elements of the full protocol.

**Supplementary Information:**

The online version contains supplementary material available at 10.1186/s13063-021-05277-7.

## Supplementary Information

**Additional file 1.** Full study protocol.

## Data Availability

The trial dataset and materials will be made publicly available via the Enlighten: Research Data repository https://www.gla.ac.uk/research/enlighten

