# Supplementary material for: The impact of theory-based messages on COVID-19 vaccination intentions: a structured summary of a study protocol for a randomised controlled trial
Source: Trials. 2021 Apr 29;22:311. doi: 10.1186/s13063-021-05277-7 (PMC8082050; doi:10.1186/s13063-021-05277-7)
Supplement: Supplementary file 1 — Additional file 1. Full study protocol. [file 13063_2021_5277_MOESM1_ESM.docx]

**Study Title:** The impact of theory-based messages on COVID-19 vaccination intentions

**Ethics Ref.:** 200200052

**Version No. and Date**: 1.0, 26^th^ February 2021

| **Chief Investigator:** | Dr Katie Robb, katie.robb@glasgow.ac.uk  University of Glasgow |
| --- | --- |
| **Investigators:** | Dr Ben Young, University of Glasgow  Dr Marie Kotzur, University of Glasgow  Ms Lauren Gatting, University of Glasgow  Prof Alex McConnachie, University of Glasgow  Prof Ronan O’Carroll, University of Stirling  Prof Kirsten McCaffery, University of Sydney  Dr Carissa Bonner, University of Sydney  Dr Julie Ayre, University of Sydney  Ms Carys Batcup, University of Sydney |
| **Sponsor:** | University of Glasgow  University Avenue, Glasgow, G12 8QQ |
| **Funder:** | University of Glasgow |

The investigators have no potential conflicts of interest to declare.

TABLE OF CONTENTS

[1. KEY TRIAL CONTACTS 3](#_Toc67994501)

[2. LAY SUMMARY 4](#_Toc67994502)

[3. SYNOPSIS 4](#_Toc67994503)

[4. ABBREVIATIONS 5](#_Toc67994504)

[5. BACKGROUND AND RATIONALE 5](#_Toc67994505)

[6. OBJECTIVES AND OUTCOME MEASURES 6](#_Toc67994506)

[7. TRIAL DESIGN 7](#_Toc67994507)

[8. PARTICIPANT IDENTIFICATION 7](#_Toc67994508)

[Trial Participants 7](#_Toc67994509)

[Inclusion Criteria 7](#_Toc67994510)

[Exclusion Criteria 7](#_Toc67994511)

[9. TRIAL PROCEDURES 7](#_Toc67994512)

[Recruitment and sampling 7](#_Toc67994513)

[Screening and Eligibility Assessment 8](#_Toc67994514)

[Informed Consent 8](#_Toc67994515)

[Randomisation 8](#_Toc67994516)

[Blinding 8](#_Toc67994517)

[Survey 8](#_Toc67994518)

[Early Discontinuation/Withdrawal of Participants 9](#_Toc67994519)

[Definition of End of Trial 9](#_Toc67994520)

[10. TRIAL INTERVENTIONS 9](#_Toc67994521)

[Intervention arm 9](#_Toc67994522)

[Control arm 9](#_Toc67994523)

[11. STATISTICS 9](#_Toc67994524)

[Description of Statistical Methods 9](#_Toc67994525)

[Pre-intervention data 9](#_Toc67994526)

[Post-intervention data 10](#_Toc67994527)

[Sample Size Determination 10](#_Toc67994528)

[Analysis Populations 10](#_Toc67994529)

[Decision Points 10](#_Toc67994530)

[Stopping Rules 11](#_Toc67994531)

[Procedure for Accounting for Missing, Unused, and Spurious Data. 11](#_Toc67994532)

[Procedures for Reporting any Deviation(s) from the Original Statistical Plan 11](#_Toc67994533)

[12. DATA MANAGEMENT 11](#_Toc67994534)

[Source Data 11](#_Toc67994535)

[Access to Data 11](#_Toc67994536)

[Data Recording and Record Keeping 11](#_Toc67994537)

[13. ETHICAL AND REGULATORY CONSIDERATIONS 12](#_Toc67994538)

[Approvals 12](#_Toc67994539)

[Participant Confidentiality 12](#_Toc67994540)

[Expenses and Benefits 12](#_Toc67994541)

[14. FUNDING 12](#_Toc67994542)

[15. PUBLICATION POLICY 12](#_Toc67994543)

[16. ARCHIVING 12](#_Toc67994544)

[17. REFERENCES 12](#_Toc67994545)

[18. APPENDIX D: AMENDMENT HISTORY 13](#_Toc67994546)

# KEY TRIAL CONTACTS

| **Chief Investigator** | Dr Katie Robb, Reader, University of Glasgow  katie.robb@glasgow.ac.uk  0141 211 0685 |
| --- | --- |
| **Investigators** | Dr Ben Young, Research Associate, University of Glasgow, ben.young@glasgow.ac.uk  Dr Marie Kotzur, University of Glasgow  Ms Lauren Gatting, University of Glasgow  Professor Alex McConnachie, , University of Glasgow  Prof Ronan O’Carroll, University of Stirling  Prof Kirsten McCaffery, University of Sydney  Dr Carissa Bonner, University of Sydney  Dr Julie Ayre, University of Sydney  Ms Carys Batcup, University of Sydney |
| **Sponsor** | University of Glasgow |
| **Funder(s)** | University of Glasgow |
| **Statistician** | Professor Alex McConnachie, Professor of Clinical Trial Biostatistics, alex.mcconnachie@glasgow.ac.uk |

# LAY SUMMARY

Uptake of a vaccine against COVID-19 is key to controlling the pandemic, but approximately three in ten people in the UK report that they do not intend to have the vaccine, often because they are concerned about its side effects or safety. This study will assess people’s intentions to receive a COVID-19 vaccine in Scotland and if theory-based messages can change beliefs and increase intentions. The messages will be based on publicly available information about the need for COVID-19 vaccination and the safety of the vaccine, and structured to address specific types of treatment beliefs. Participants will be put at random into either a group that are shown the messages or another group that are shown general information about the COVID-19 virus and vaccination programme. By comparing the two groups we aim to test if the theory-based messages have change people’s intention to get a COVID-19 vaccine, understanding and perceived necessity of COVID-19 vaccination and concerns about the COVID-19 vaccines. The findings will allow planners of Scotland’s vaccination programme to predict how many people will want to be vaccinated in different regions in Scotland, and provide evidence of the effect of theory-based health messages on people’s intentions and beliefs about COVID-19 vaccination.

# SYNOPSIS

| Trial Title | The impact of theory-based messages on Covid-19 vaccination intentions | | |
| --- | --- | --- | --- |
| Internal ref. no. (or short title) | Messaging for Covid-19 vaccination | | |
| Trial Registration | ClinicalTrials.gov NCT04813770 | | |
| Sponsor | University of Glasgow | | |
| Funder | University of Glasgow | | |
| Trial Design | Prospective, parallel two-arm randomised controlled trial | | |
| Trial Participants | Adults living in Scotland | | |
| Sample Size | 1094 | | |
| Planned Trial Period | Total length of the project = 1 month  Duration of an individual participant’s involvement (intervention phase and all follow up) = 10 minutes | | |
| Planned Recruitment Period | 31^st^ March 2021 to 30^th^ April 2021 | | |
|  | Objectives | Outcome Measures | Timepoint(s) |
| Primary | Assess the impact of theory-based messages on COVID-19 vaccination intention. | Self-reported intention to receive a vaccine for COVID-19 if invited. | Immediately post-intervention. |
| Secondary | Assess the impact of theory-based messages on understanding of COVID-19, beliefs and concerns about COVID-19 vaccination, and whether those variables mediate change in vaccination intention. | Self-reported COVID-19 illness coherence, perceived necessity of a COVID-19 vaccine and concerns about a COVID-19 vaccine. | Immediately post-intervention. |
|  | Assess baseline intentions to be vaccinated for COVID-19 in Scotland, prevalence of beliefs about COVID-19 vaccination, and characteristics and beliefs associated with baseline intentions. | Self-reported beliefs about COVID-19 vaccination and intention to receive a vaccine for COVID-19 if invited. | Immediately pre-intervention. |
| Intervention(s) | Brief exposure to messages addressing necessity beliefs and concerns about COVID-19 vaccination. | | |
| Comparator | Brief exposure to messages containing general information about COVID-19 and COVID-19 vaccination. | | |

# ABBREVIATIONS

| BMQ | Beliefs about Medicines Questionnaire |
| --- | --- |
| Brief IPQ | Brief Illness Perceptions Questionnaire |
| COVID-19 | SARS-CoV-2 / Severe acute respiratory syndrome coronavirus 2 |
| GDPR | General Data Protection Regulation |
| IPQ-R | Revised Illness Perceptions Questionnaire |

# BACKGROUND AND RATIONALE

The novel coronavirus SARS-CoV-2 (COVID-19) has been associated with over 2 million deaths worldwide ^1^. Vaccination will play an important role in controlling the COVID-19 pandemic and reducing the need for non-pharmaceutical interventions such as lockdowns and social distancing. Candidate COVID-19 vaccines have progressed at speed through stages of development, evaluation and regulatory approval ^2^. However, the success of a vaccination programme relies upon high uptake of a vaccine in addition to its effectiveness.

The UK vaccination programme aims initially to reduce symptom severity in those who contract the virus ^2^. Communication about the necessity of a vaccine is therefore paramount. Groups at highest risk of COVID-19 mortality are prioritised in the roll-out of approved vaccines, predominantly targeting adults aged over 50 years ^3^.

There is evidence from multiple studies that only between 64-73% of the UK population are willing to receive a COVID-19 vaccination, including those at increased risk of COVID-19 mortality. Similar acceptance levels have been reported in other countries including the US, Italy and Spain ^4-8^. With variations in how the UK nations have responded to the pandemic, and likely associated differences in public confidence and trust ^9^, there is a need to assess vaccination intentions in a national population sample in Scotland.

In an Australian study, individuals with inadequate health literacy experienced more difficulty understanding government messaging about COVID-19, and were more likely to endorse vaccination misinformation beliefs than people with adequate health literacy ^10^. This highlights an important target group for COVID-19 vaccination messaging to achieve high uptake in the overall population.

There has been a call for research into behavioural interventions to promote adherence to COVID-19 preventative behaviours including vaccination uptake ^11^. Research into medication adherence has found that sociodemographic and clinical factors are not good predictors of adherence, whereas beliefs about illness and treatment are established as independent predictors ^12^. Research suggests that barriers to COVID-19 vaccination centre around beliefs about the consequences of the vaccine for oneself and others, involving themes of personal health, health consequences to others, concerns of vaccine safety, and severity of COVID-19 ^13^. Specific concerns about vaccine safety involve beliefs that the ‘rushed’ vaccine could cause side effects or be unsafe ^13, 14^.

Adherence to social distancing and lockdown guidelines for COVID-19 was found in a survey to be associated with perceived illness coherence and perceived necessity of the behaviour to prevent the spread of COVID-19 ^15^. Perceived coherence in this context involves an understanding of COVID-19 in a way that is felt to be useful and makes sense. Individuals have reported uncertainty about key COVID policy issues, particularly where there has been an evolving and contested scientific viewpoint ^16^.

Providing information to address stroke survivors’ beliefs about medication and stroke, in conjunction with an implementation intentions plan, increased necessity beliefs relative to medication concerns and improved adherence to medication by 10% ^17^. This approach is based on Leventhal's Self-Regulation Model which proposes that individuals hold a ‘common sense representation’ of an illness based on a range of illness perceptions ^18^. An extension to this model, the necessity-concerns framework, incorporates beliefs and concerns about medical treatment ^19^. Given the importance of perceived coherence and necessity in COVID-19 preventative behaviours, and evidence of the effectiveness of the necessity-concerns approach in medication adherence, this strategy has potential to improve uptake of a COVID-19 vaccine.

We aim to i) measure intentions to be vaccinated for COVID-19 in Scotland and ii) evaluate the impact on COVID-19 vaccination intentions and beliefs of theory-based messages that aim to simultaneously increase the perceived necessity of a COVID-19 vaccination and reduce perceived concerns about a COVID-19 vaccination.

# OBJECTIVES AND OUTCOME MEASURES

| **Objectives** | **Outcome Measures** | **Timepoint of evaluation of this outcome measure** |
| --- | --- | --- |
| **Primary Objective** Assess the impact of theory-based messages on COVID-19 vaccination intention. | Self-reported intention to receive a vaccine for COVID-19 if invited. | Immediately post-intervention. |
| **Secondary Objectives** Assess the impact of theory-based messages on understanding of COVID-19, and beliefs and concerns about COVID-19 vaccination, and whether those variables mediate change in vaccination intention. | Self-reported COVID-19 illness coherence, perceived necessity of a COVID-19 vaccine and concerns about a COVID-19 vaccine. | Immediately post-intervention. |
| To assess baseline COVID-19 vaccine acceptance levels and barriers in Scotland. | Self-reported intention to receive a vaccine for COVID-19 if invited.  Self-reported barriers to receiving a vaccine for COVID-19. | Immediately pre-intervention. |

# TRIAL DESIGN

Prospective, parallel two-arm individually randomised controlled trial. Online intervention with pre- and post-data collection via an online questionnaire. Screening, treatment, and post-treatment follow-up in a single session.

# PARTICIPANT IDENTIFICATION

## Trial Participants

Individuals living in Scotland aged over 18, with a focus on individuals with lower levels of formal education.

## Inclusion Criteria

- Willing and able to give informed consent for participation in the trial.
- Aged 18 years or above.
- Resident in Scotland.

## Exclusion Criteria

- Received at least one COVID-19 vaccination dose.

# TRIAL PROCEDURES

## Recruitment and sampling

Individuals will be identified and approached by a social research agency (‘the agency’). They will have previously provided their consent to take part in research by signing up to an existing participant panel (‘the panel’). Individuals will be approached with the aim of achieving a sample with at least 60% with either no educational qualifications or school-level qualifications (e.g. National 5s/Standard Grades). This will involve a quota sampling approach based on sociodemographic characteristics (age, gender, ethnic group, education and occupation), with regular review of quotas and over-sampling of individuals with underrepresented characteristics.

## Screening and Eligibility Assessment

All individuals will be screened for eligibility by providing their age group, location and COVID-19 vaccination status. Individuals aged under 18 years, not living in Scotland or already vaccinated for COVID-19 will be excluded at this stage.

## Informed Consent

Individuals will be presented with a simple electronic participant information page describing the nature of the research. Participants will provide informed consent via a digital tick box and confirmation screen prior to any study procedures being undertaken. It will be clearly stated that the participant is free to withdraw at any time for any reason and with no obligation to give the reason for withdrawal.

The participant will be allowed as much time as they wish to consider the information, and the opportunity to question the Investigator to decide whether they will participate in the study.

## Randomisation

Participants will be allocated to one of two groups. Quasi-randomisation will be performed automatically by the online survey platform by creating a variable derived from the number of seconds in the minute that the participant initiates the survey. Participants starting the survey at 0-14 or 30-44 seconds in the minute will be allocated to the intervention arm and 15-29 or 45-59 seconds to the control arm.

## Blinding

Participants will not be blinded to the assigned intervention but will not be informed of the purpose of the study until they have completed the follow-up survey, at which point they will be debriefed. Investigators will be blinded to allocation because all procedures will be undertaken digitally and remotely without any investigator contact with participants.

## Survey

All participants will complete an anonymous survey before and after the intervention.

Pre-intervention survey: COVID-19 vaccination intention; beliefs about COVID-19 and COVID-19 vaccination; barriers to COVID-19 vaccination.

Post-intervention survey: COVID-19 vaccination intention; beliefs about COVID-19 and COVID-19 vaccination; health literacy; COVID-19 behaviours; sociodemographic characteristics.

To assess vaccination intention participants will be asked: "If you were invited to have a COVID-19 vaccination would you take the vaccine?"

Secondary outcome measures include the Brief Illness Perception Questionnaire (Brief IPQ), the illness coherence subscale of the Revised Illness Perceptions Questionnaire (IPQ-R) ^20^ and the Specific-Necessity and Specific-Concerns subscales of the Beliefs about Medicines Questionnaire (BMQ) ^21^.

## Early Discontinuation/Withdrawal of Participants

During the course of the survey a participant may choose to stop taking part or withdraw their consent at any time. By closing their browser, they can stop taking part but permit data obtained up until the point of withdrawal to be retained for use in the study analysis. No further data would be collected after withdrawal. By contacting the agency or the panel, they can withdraw consent and withdraw the data collected up until the point of withdrawal.

## Definition of End of Trial

The end of trial is the point at which all the data have been entered and queries resolved.

# TRIAL INTERVENTIONS

## Intervention arm

The intervention arm participants will be asked to look at theory-based health messages about COVID-19 and COVID-19 vaccination, the necessity of COVID-19 vaccination to oneself and others, and COVID-19 vaccine regulatory approval processes. These messages are based on publicly available information and are hypothesised to increase perceived necessity and reduce concerns about vaccination, and target known barriers to vaccine uptake.

## Control arm

The control arm participants will be asked to look at general messages about the COVID-19 virus and the vaccination programme. Those messages do not target necessity and concerns, but are anticipated to promote understanding of the pandemic.

The messages for both arms are of similar length, format and readability. Both sets of messages will be accompanied by images that relate to the messages, for example photographs of a hospital waiting room, a scientist or doctor, or a person being vaccinated.

Participants will be asked to look at the messages on their screen for as long as they wish. There will be no minimum message exposure time and they will have the freedom to proceed through the message content at a pace of their choosing.

# STATISTICS

The plan for the statistical analysis of the trial is outlined below. There is not a separate SAP document in use for the trial.

## Description of Statistical Methods

### Pre-intervention data

Participant characteristics and pre-intervention intentions to be vaccinated will be described with frequencies and percentages, and compared between treatment arms using Pearson's χ2 tests. Intentions will be dichotomised with positive responses (Yes, definitely; Yes, probably) recoded as intenders and other responses (Don’t know; Probably not; Definitely not) as non-intenders and analysed by participant characteristics (e.g. age group, region, health literacy) using Pearson's χ2 tests.

Beliefs about COVID-19 and vaccination measured using validated scales (Brief IPQ, IPQ-R and BMQ) will have scores calculated as per the recommended guidance for the respective measures, and summarised as frequencies or means.

Other beliefs about COVID-19 and vaccination will be dichotomised as agreement (Agree/Strongly agree) or non-agreement (Don’t know/Probably not/Definitely not). Scores will first be reversed for some items as appropriate. Data will then be summarised as frequencies and percentages.

To identify characteristics and beliefs associated with intention to be vaccinated (intender/non-intender), logistic regression will be used with independent variables representing sociodemographic (e.g. age; socioeconomic group; health literacy), clinical (e.g. vulnerability to COVID-19) and psychological (e.g. belief that the COVID‐19 outbreak is going to continue for a long time; belief the risks of COVID‐19 have been exaggerated; concerns about vaccine safety). Due to a large number of predictors, statistical significance will be set at p < 0.01.

### Post-intervention data

The primary outcome is intention to be vaccinated immediately post-intervention. Responses will be dichotomised as intender/non-intender as described above. The outcome will be analysed on an intention-to-treat basis using logistic regression and a p value of 0.05. There will be no adjustment for multiplicity because a clear primary outcome is defined and all other outcomes serve as secondary investigations.

Secondary outcomes are beliefs about vaccination and the disease. Scores will be recoded and analysed by following the recommended procedures for the respective measures used. The IPQ-R coherence subscale, and each subscale of the BMQ, produces a score in the range 5-25. The Brief IPQ generates a score in the range 0-80. Difference in mean scores between groups will be analysed using independent samples t-tests if scores are normally distributed or Mann-Whitney U tests if distributions are skewed.

Logistic regression will be used to test for mediation of the relationship between group allocation and intention to be vaccinated by change in the hypothesised mechanisms (perceived necessity; perceived concerns; illness coherence).

## Sample Size Determination

The sample size of 1094 will provide 80% power to detect a 7.2% absolute increase in the proportion of participants intending to be vaccinated for COVID-19, assuming a baseline proportion of 73% and a 0.05 significance level.

## Analysis Populations

All participants as randomised (intention to treat).

## Decision Points

None.

## Stopping Rules

There are no stopping criteria because the data will be collected over a short timeframe, due to the use of an established research panel who are receptive to survey invitations. We will make recommendations to Public Health teams if the effect size favours the intervention arm at the end of the study.

## Procedure for Accounting for Missing, Unused, and Spurious Data.

There will be no missing data for primary or secondary outcomes as participants are required to enter a response to all questions in order to complete the survey.

Where participants select ‘prefer not to say’ for sociodemographic characteristics the data will be reported as missing.

## Procedures for Reporting any Deviation(s) from the Original Statistical Plan

Any deviation from the original statistical plan will be described and justified in the final report.

# DATA MANAGEMENT

The plan for the data management of the study is outlined below. There is not a separate Data Management document in use for the trial.

## Source Data

Source data are the participants’ responses to survey questions, which form an anonymous electronic database. All data will be stored securely at all times.

## Access to Data

Direct access will be granted to authorised representatives from the Sponsor, host institution and the regulatory authorities to permit trial-related monitoring, audits and inspections.

In keeping with open access principles, other researchers can request access to the study data.

## Data Recording and Record Keeping

All trial data will be entered by participants on web-based forms via an internet browser, managed by the agency and the panel. Participants will be provided with a unique identification number. Any identifiable information held for the administration of the panel will be stored separately by the agency or panel. Once the trial is complete, the data file will be password protected by the agency and transferred securely to the researchers either via the University of Glasgow File Transfer Service or DataXchange. The data received by the researchers will not include any personally identifiable information. Any identifiable information will be omitted in data transfer between the panel to the agency, and the agency to the researchers.

Data will be retained for 10 years in accordance with university policy.

# ETHICAL AND REGULATORY CONSIDERATIONS

## Approvals

The study procedures, informed consent form, participant information page, intervention messages and survey were approved by the University of Glasgow College of MVLS Research Ethics Committee, ref. 200200052, on 29^th^ January 2021.

The Investigator will submit and, where necessary, obtain approval for proposed changes to the approved documents.

## Participant Confidentiality

The study constitutes a single anonymous survey and will not collect participant names, dates of birth or contact details. The study will therefore comply with the GDPR and Data Protection Act 2018, which require data to be de-identified as soon as it is practical to do so. The name and any other identifying data will not be included in any trial data electronic file. The participants will be identified by a unique trial specific number and/or code in any database.

All documents will be stored securely and only accessible by study staff and authorised personnel. The panel and agency will safeguard the privacy of participants’ personal data. The researchers will not be supplied with any personally identifiable data by the agency.

## Expenses and Benefits

Participants will receive a small monetary reward for completing the survey.

# FUNDING

The study is funded by the University of Glasgow USyd-Glasgow Partnership Collaboration Award.

# PUBLICATION POLICY

Study findings will be disseminated in academic journals, scientific meetings and with vaccination programme co-ordinators.

# ARCHIVING

The study will be archived for 10 years.

# REFERENCES

1. Center for Systems Science and Engineering (CSSE) at Johns Hopkins University. COVID-19 Dashboard. 2020; Available from: <https://coronavirus.jhu.edu/map.html>.

2. Bingham K. The UK Government's Vaccine Taskforce: strategy for protecting the UK and the world. The Lancet. 2020; (Online first).

3. Joint Committee on Vaccination and Immunisation. Updated interim advice on priority groups for COVID-19 vaccination. 2020; Available from: <https://www.gov.uk/government/publications/priority-groups-for-coronavirus-covid-19-vaccination-advice-from-the-jcvi-25-september-2020>.

4. Pogue K, Jensen JL, Stancil CK, Ferguson DG, Hughes SJ, Mello EJ, et al. Influences on Attitudes Regarding Potential COVID-19 Vaccination in the United States. Vaccines (Basel). 2020; 8.

5. Malik AA, McFadden SM, Elharake J, Omer SB. Determinants of COVID-19 vaccine acceptance in the US. EClinicalMedicine. 2020; 26.

6. Gallup. One in Three Americans Would Not Get COVID-19 Vaccine. 2020; Available from: <https://news.gallup.com/poll/317018/one-three-americans-not-covid-vaccine.aspx>.

7. Lazarus JV, Ratzan SC, Palayew A, Gostin LO, Larson HJ, Rabin K, et al. A global survey of potential acceptance of a COVID-19 vaccine. Nature Medicine. 2020.

8. Pew Research Center. Most Americans expect a COVID-19 vaccine within a year; 72% say they would get vaccinated. 2020; Available from: <https://www.pewresearch.org/fact-tank/2020/05/21/most-americans-expect-a-covid-19-vaccine-within-a-year-72-say-they-would-get-vaccinated/>.

9. Fancourt D, Bu F, WanMak H, Steptoe A. Covid-19 Social Study - Results Release 22. 2020; Available from: <https://b6bdcb03-332c-4ff9-8b9d-28f9c957493a.filesusr.com/ugd/3d9db5_636933e8191d4783866c474fab3ca23c.pdf>.

10. McCaffery K, Dodd RH, Cvejic E, Ayre J, Batcup C, Isautier JM, et al. Disparities in COVID-19 related knowledge, attitudes, beliefs and behaviours by health literacy. medRxiv. 2020:2020.06.03.20121814.

11. Arden MA, Byrne-Davis L, Chater A, Hart J, McBride E, Chilcot J. The vital role of health psychology in the response to COVID-19. British Journal of Health Psychology. 2020; 25:831-8.

12. Horne R, Weinman J. Self-regulation and Self-management in Asthma: Exploring The Role of Illness Perceptions and Treatment Beliefs in Explaining Non-adherence to Preventer Medication. Psychology & Health. 2002; 17:17-32.

13. Williams L, Gallant AJ, Rasmussen S, Brown Nicholls LA, Cogan N, Deakin K, et al. Towards intervention development to increase the uptake of COVID-19 vaccination among those at high risk: Outlining evidence-based and theoretically informed future intervention content. British Journal of Health Psychology. 2020; 25:1039-54.

14. Sherman SM, Smith LE, Sim J, Amlôt R, Cutts M, Dasch H, et al. COVID-19 vaccination intention in the UK: Results from the COVID-19 Vaccination Acceptability Study (CoVAccS), a nationally representative cross-sectional survey. medRxiv. 2020:2020.08.13.20174045.

15. UK COVID-19 Mental Health and Wellbeing study (UK COVID-MH). 2020.

16. The Policy Institute Kings College London. Coronavirus uncertainties: vaccines, symptoms and contested claims. 2020; Available from: <https://www.kcl.ac.uk/policy-institute/assets/coronavirus-uncertainties.pdf>.

17. O'Carroll RE, Chambers JA, Dennis M, Sudlow C, Johnston M. Improving adherence to medication in stroke survivors: a pilot randomised controlled trial. Ann Behav Med. 2013; 46:358-68.

18. Leventhal H, Diefenbach M, Leventhal EA. Illness cognition: Using common sense to understand treatment adherence and affect cognition interactions. Cognitive Therapy and Research. 1992; 16:143-63.

19. Horne R, Chapman SC, Parham R, Freemantle N, Forbes A, Cooper V. Understanding patients' adherence-related beliefs about medicines prescribed for long-term conditions: a meta-analytic review of the Necessity-Concerns Framework. PLoS One. 2013; 8:e80633.

20. Moss-Morris R, Weinman J, Petrie K, Horne R, Cameron L, Buick D. The Revised Illness Perception Questionnaire (IPQ-R). Psychology & Health. 2002; 17:1-16.

21. Horne R, Weinman J, Hankins M. The beliefs about medicines questionnaire: The development and evaluation of a new method for assessing the cognitive representation of medication. Psychology & Health. 1999; 14:1-24.

# APPENDIX D: AMENDMENT HISTORY

| **Amendment No.** | **Protocol Version No.** | **Date issued** | **Author(s) of changes** | **Details of Changes made** |
| --- | --- | --- | --- | --- |
|  |  |  |  |  |
